# Diversity in substance use behaviour among street children of Delhi under Bayesian paradigm

**DOI:** 10.1186/s12874-020-01172-y

**Published:** 2020-12-01

**Authors:** Vivek Verma, Ashwani Kumar Mishra, Anju Dhawan, Dilip C. Nath

**Affiliations:** 1 Department of Neurology, All India Institute of Medical Sciences (AIIMS), New Delhi, India; 2 National Drug Dependence Treatment Centre (NDDTC), All India Institute of Medical Sciences (AIIMS), New Delhi, India; 3 Assam University, Silchar, Assam, India

**Keywords:** Response driven sampling, Street children, Shannon diversity index, Prior distribution, Posterior distribution

## Abstract

**Background:**

Shannon’s index is one of the measures of biodiversity, which is intended to quantify both richness and evenness of the species/individuals in the ecosystem or community. However, application of Shannon’s index in the field of substance use among the street children has not been done till date.

**Methods:**

This paper is concerned with methods of estimating Shannon’s diversity index (SDI), which can be used to capture the variation in the population due to certain characteristics. Under the consideration that the probability of abundance, based on certain characteristics in the population, is a random phenomenon, we derive a Bayesian estimate in connection with Shannon’s information measure and their properties (mean and variance), by using a probability matching prior, through simulation and compared it with those of the classical estimates of Shannon. The theoretical framework has been applied to the primary survey data of substance use among the street children in Delhi, collected during 2015. The measure of diversity was estimated across different age profiles and districts.

**Results:**

The results unrevealing the diversity estimate for street children corresponding to each region of Delhi, under both the classical and Bayesian paradigms. Although the estimates were close to one another, a striking difference was noted in the age profile of children.

**Conclusions:**

The Bayesian methodology provided evidence for a greater likelihood of finding substance-using street children, belonging to the lower age group (7-10, maximum Bayesian entropy-3.73), followed by the middle (11-14) and upper age group (15-18). Moreover, the estimated variance under the Bayesian paradigm was lesser than that of the classical estimate. There is ample scope for further refinement in these estimates, by considering more covariates that may have a possible role in initiating substance use among street children in developing countries like India.

## Background

In ecology, a diversity index is a parameter of interest intended to measure biodiversity within an ecosystem [1]. The widely used diversity indices are Shannon’s index and Simpson’s index. They can be used to assess the diversity of any population in which each member belongs to a unique species. Shannon’s diversity index (SDI) has been derived from the information theory, originally in the context of information in telephone systems. The SDI measures enables in ascertaining the membership of species chosen randomly to a particular class from the collection of all possible classes. This average uncertainty increases as the number of species increases. It attains maximum as the relative abundances of species are equal. A diversity index is a mathematical measure of species diversity in a community. Diversity indices provide more information about community composition than simply species richness (i.e., the number of species present); they also take the relative abundances of different species into account. The term diversity although seems to be terminology of an ecosystem, its analogous statistical terminology is dispersion. Hence, application of a diversity index can be reviewed as a mathematical measure of species or a particular community under investigation, which facilitates precise delimitation of community composition,which can be characterized by the statistical measures of Mean and Variance.

In earlier studies, estimation of SDI using various techniques viz., the maximum likelihood estimator [2], jackknife method [3], combined Horvitz-Thompson estimator [4] and sample coverage [5], were discussed by various authors. SDI [6] has two properties: (i) Shannon’s index is zero if and only if there is one specie in the community, and (ii) Shannon’s index is maximum only when all species are present within a studied ecosystem with the same probabilities. Typically the values lies between 1.5 and 3.5 in most ecological studies [7, 8], and the index is rarely greater than 4. SDI increases as both the richness and the evenness of the community increase. The measure greater than 1 implies more diversity and thereby greater likelihood of finding more species or individuals with characteristics under investigation, for example in this study substance using street children. Higher the value of indices more is the richness in terms of the species/individuals.

In the present article, we derived a probability matching prior for SDI under Bayesian paradigm, and demonstrated the relevance of posterior estimate through numerical simulation and real-life application, and compared with classical estimate of SDI. The primary data used for the study was collected during August, 2015 to November, 2015 through a representative survey by implementing a Respondent Driven Sampling (RDS) among street children in all nine districts of Delhi. The suggestive methodology is used to demonstrate the derivable benefits in terms of better characterization of age profile and variation across districts of substance using street children in Delhi.

## Methods

The methodology of the present article has been elaborated by mathematical formulation of SDI, under the classical and Bayesian paradigm. Under both suggested analytical approaches, the derivations of mean and variance have been used to demonstrate its performance for describing the diversity in population with certain characteristic. The comparative performance of these estimators has been evaluated and presented, by applying the methodology on real life based data of substance using children in Delhi.

### Mathematical formulation of SDI under classical paradigm

Suppose that there are *S* number of different species in a community and *S*, is assumed to be a known quantity and is relatively small. Let *p*_*i*_ denotes the probability of richness of the *i*^*th*^ species in the community, for all *i*=1,2,⋯,*S* and is such that $\sum \limits _{i=1}^{S}p_{i}=1$. One of the widely used mathematical measures of species diversity in a ecological community is the SDI, which is the linear function of probabilities, *p*_1_,*p*_2_,⋯*p*_*s*_, defined as

1 $$\begin{array}{@{}rcl@{}}  H(\boldsymbol{p})=-\sum\limits_{i=1}^{s}p_{i} \ log(p_{i}). \end{array} $$

Suppose that a random sample of *n* has been collected and corresponding to each selected unit let *X*_*ij*_ is an indicator function which takes value ‘1’ if the *j*^*th*^ selected unit belonging to *i*^*th*^ species and ‘0’ otherwise, for all *j*=1,2,⋯,*n* and *i*=1,2,⋯,*S*. Let us define the variables $Y_{i}=\sum \limits _{j=1}^{n}X_{ij}$, denoting the number of sampled units belongs *i*^*th*^ species in the community. Further suppose that, *X*_1_,*X*_2_,⋯,*X*_*S*_ are independently distributed as *Y*_*i*_∼*Binomial*(*n*,*p*_*i*_). Under the assumption that the parameter *p*_*i*_, the probability of abundance of the *i*^*th*^ species in the community, is an unknown but a fixed quantity, then by following the maximum likelihood principle *p*_*i*_ is estimated as $\hat {p_{i}}=\frac {x_{i}}{n}$. Based on the estimated *p*_*i*_, say $\hat {p_{i}}$, the mean and variance of SDI, *H*(***p***), have obtained as

2 $$ \begin{aligned} \hat{H}(\boldsymbol{p})=E(H(\boldsymbol{p}))&=-E\left[ \sum\limits_{i=1}^{s}p_{i} \ log(p_{i})\right]\\&=-E\left[\sum\limits_{i=1}^{s}\frac{x_{i}}{n} \ log\left(\frac{x_{i}}{n}\right)\right]\\ &=\frac{-1}{n}E\left[\sum\limits_{i=1}^{s}x_{i} \left(log \ x_{i}-log \ n\right)\right] \\&=\frac{-1}{n}\sum\limits_{i=1}^{s} E\left(x_{i} \log \ x_{i}- x_{i}\ log \ n\right)\\ &=\frac{-1}{n}\sum\limits_{i=1}^{s} \left(E(x_{i} \log \ x_{i})- np_{i}\ log \ n\right). \end{aligned}  $$

3 $$\begin{array}{@{}rcl@{}} V\left(\hat{H}(\boldsymbol{p})\right)&=&V\left[ \sum\limits_{i=1}^{s}p_{i} \ log(p_{i})\right] =V\left[\sum\limits_{i=1}^{s}\frac{x_{i}}{n} \ log\left(\frac{x_{i}}{n}\right)\right] \\ &=&\frac{1}{n^{2}}V\left[\sum\limits_{i=1}^{s}x_{i} \left(log \ x_{i}-log \ n\right)\right] \\&=&\frac{1}{n^{2}}\sum\limits_{i=1}^{s} V\left(x_{i} \log \ x_{i}- x_{i}\ log \ n\right) \\ &=&\frac{1}{n^{2}}\sum\limits_{i=1}^{s} \left(V(x_{i} \log \ x_{i})+np_{i}(1\,-\,p_{i})\ (log \ n)^{2}\right).\\ \end{array} $$

Let *g*(*X*_*i*_)=*x*_*i*_ log(*x*_*i*_) and *p*_*i*_’s are independent, then the *E*(*g*(*X*_*i*_)) and *V*(*g*(*X*_*i*_)) have derived using the Delta method [9] as

4 $$\begin{array}{@{}rcl@{}} E\left(g(X_{i})\right)&\approx&g\left(E(X_{i})\right)+\frac{1}{2}g^{\prime\prime}\left(E(X_{i})\right)V(X_{i}) \\ &=& np_{i} \ log(np_{i}) +\frac{(1-p_{i})}{2}, \end{array} $$

5 $$\begin{array}{@{}rcl@{}} V\left(g(X_{i})\right)&\approx&\left[g'\left(E(X_{i})\right)\right]^{2}V(X_{i})\\&=& [1+log(np_{i})]^{2} \ np_{i}(1-p_{i}). \end{array} $$

Using the above equations, the *E*(*H*(***p***)) and *V*(*H*(***p***)) is given by

6 $$\begin{array}{@{}rcl@{}} E(H(\boldsymbol{p}))\!\!&=&\frac{-1}{n}\sum\limits_{i=1}^{s} \left(np_{i} \ log(np_{i}) +\frac{(1-p_{i})}{2}- np_{i}\ log \ n\right) \\ \!\!&=&-\sum\limits_{i=1}^{s} p_{i} \left(log(np_{i}) +\frac{(1-p_{i})}{2n}- \ log \ n\right) \end{array} $$

7 $$\begin{array}{@{}rcl@{}} V(H(\boldsymbol{p}))&=&\frac{1}{n^{2}}\sum\limits_{i=1}^{s} \left([1+log(np_{i})]^{2} \ np_{i}(1-p_{i})\right.\\&&\quad+\left.np_{i}(1-p_{i})\ (log \ n)^{2}\right) \\ &=&\frac{1}{n}\sum\limits_{i=1}^{s} p_{i}(1-p_{i}) \left(\left[1+log(np_{i})\right]^{2}\right.\\&&\quad+\left.(log \ n)^{2}\right) \end{array} $$

### Mathematical formulation of SDI under Bayesian paradigm

In the previous section, the usual estimation procedure of SDI, *H*(***p***), has been discussed, which is based on the assumption that the probability of abundance of the *i*^*th*^ species in the community, *p*_*i*_, is an unknown but a fixed quantity. But in practical situations *p*_*i*_’s might be a random quantity, and hence under this situation the randomness of SDI, *H*(***p***), can be quantified under Bayesian paradigm by specifying a suitable prior distribution for *H*(***p***). To suggest a prior distribution for a function of *p*_*i*_’s, *H*(***p***), is difficult to obtain directly. Here, we derived a probability matching prior, distribution for *H*(***p***) for a discrete binary population, which is free from any hyper-parameter. In principle, a probability matching prior holds the promise of providing a possible agreement between frequentist and Bayesian inferential procedures, which can be used for routine use in Bayesian inference [10, 11].

#### **Theorem 1**

Suppose *q*(·) defines the prior distribution for $H(\boldsymbol {p})=-\sum \limits _{i=1}^{S}p_{i} \ log(p_{i})$, a linear function of probabilities of abundance of different species in the community, then *p*_1_,⋯,*p*_*i*_,⋯*p*_*S*_, takes the following distributional form

8 $$ {}\begin{aligned} q(\boldsymbol{p})&=q(p_{1},\cdots,p_{c})\propto \left\lbrace\sum\limits_{i=1}^{S}\left(1+log(p_{i})\right)^{2} \ p_{i}(1-p_{i})\right\rbrace^{\frac{1}{2}}\\&\quad \prod\limits_{i=1}^{S} \left[p_{i}\left(1-p_{i}\right)\left(1+log(p_{i})\right)\right]^{-1}. \end{aligned}  $$

#### *Proof*

The proof of Theorem 1 is given in the Appendix. □

The posterior distribution of ***p*** for the given information will then be

9 $$\begin{array}{@{}rcl@{}} h(\boldsymbol{p}|data) &\propto& L(\boldsymbol{p}|data)\ q(\boldsymbol{p}) \\  h(\boldsymbol{p}|\boldsymbol{x})&\propto& \left\lbrace\sum\limits_{i=1}^{S}\left(1+log(p_{i})\right)^{2} \ p_{i}(1-p_{i})\right\rbrace^{\frac{1}{2}}\\&& \quad\prod\limits_{i=1}^{S} \left[p_{i}^{x_{i}}(1-p_{i})^{n-x_{i}}\left(1+log(p_{i})\right)\right]^{-1}.\\ \end{array} $$

Here, the posterior distribution, *h*(***p***|***x***), does not have any explicit form and *p*_1_,⋯,*p*_*S*_ are independent, for this reason one has to get samples from *h*(***p***|***x***) to get the posterior distribution of *H*(***p***). This is done by simulating *N* values from the posterior distribution as $\{p^{(t)}_{1},p^{(t)}_{2}\cdots,p^{(t)}_{S}; \ t= 1,2,\cdots,N\}$, then by computing *H*^(1)^(***p***),*H*^(2)^(***p***),⋯,*H*^(*N*)^(***p***), where $H^{(t)}(\boldsymbol {p})=-\sum \limits _{i=1}^{S} p^{(t)}_{i}log(p^{(t)}_{i})$. The procedure of Markov Chain Monte Carlo (MCMC) simulation technique is adopted to estimate the posterior mean and variance of *H*(***p***) and can be approximated as

10 $$\begin{array}{@{}rcl@{}} \hat{H}(\boldsymbol{p})^{B}=E(H(\boldsymbol{p})|\boldsymbol{x}) \simeq \frac{1}{N}\sum\limits_{t=1}^{N} H^{(t)}(\boldsymbol{p}) \end{array} $$

and

11 $$\begin{array}{@{}rcl@{}} V\left(\hat{H}(\boldsymbol{p})^{B}\right)&=& V(H(\boldsymbol{p})|\boldsymbol{x})\simeq\frac{1}{N}\sum\limits_{t=1}^{N}\left[H^{(t)}(\boldsymbol{p})\right]^{2}\\&&\quad-\left[\frac{1}{N}\sum\limits_{t=1}^{N}H^{(t)}(\boldsymbol{p})\right]^{2}. \end{array} $$

## Numerical study

In the present section, we illustrate the proposed procedures numerically through a simulation study. For demonstration purpose we here considered that the probability of abundance, say *p* lies between {(0.1,0.2),(0.3,0.5), (0.6,0.8)}, we vary the number of species in the community *S*=2,3,4, and number of collected samples *n*=10,50,100. For different combinations of *S*,*p*,*n*, both classical and posterior estimates (*H*(***p***) and *H*(***p***)^*B*^) and their respective variances are computed. Corresponding to each of these combinations we computed both the classical and Bayesian estimates of SDI and reported through Table 1. Here, we computed the posterior estimates through MCMC simulation technique with the help of Metropolis-Hasting’s algorithm (given in Appendix). The posterior estimate of SDI is obtained using simulation of *N*=500,000 samples, with burn-in period 10000, which is minimum number of sample required for the Markov Chain to reach stationarity. The obtained result under Table 1 shows that with increasing sample size, say *n*, under both the classical and Bayesian estimates of SDI got closer to one another, irrespective of number of species(*n*) and probability of abundance(*p*). Even with closer of estimated values of SDI, the posterior variance was detected to be significantly lower than that of the variance obtained under classical paradigm. In addition to that for all combinations of *S*;*p*;*n*, two estimators are more or less equally but the posterior variance of SDI under Bayesian set-up is again found to be significantly lower than that of classical variance of SDI.

**Table 1:** Classical and Bayesian estimates of Shannon diversity index (SDI)

S	*p*	n	$\hat {H}(\boldsymbol {p})$				$\hat {H}^{B}(\boldsymbol {p})$				$V\left (\hat {H}(\boldsymbol {p})\right)$				$V\left (\hat {H}^{B}(\boldsymbol {p})\right)$			
			*S*				*S*				*S*				*S*			
			1	2	3	4	1	2	3	4	1	2	3	4	1	2	3	4
2	(0.1,0.2)	10	1.37	1.53			1.86	1.86			1.16	1.19			0.040	0.030		
	(0.3,0.5)	10	1.76	1.88			1.78	1.94			1.17	1.05			0.046	0.014		
	(0.6,0.8)	10	1.96	1.90			2.01	2.02			0.96	1.01			0.007	0.007		
	(0.1,0.2)	50	1.82	1.90			1.77	1.94			1.06	0.84			0.040	0.010		
	(0.3,0.5)	50	1.96	1.97			2.04	2.02			0.69	0.57			0.003	0.002		
	(0.1,0.2)	100	1.93	1.95			2.05	1.99			0.81	0.63			0.008	0.003		
3	(0.1,0.2)	10	1.73	2.09	0.76		2.54	2.67	2.45		1.14	1.18	0.74		0.147	0.011	0.160	
	(0.3,0.5)	10	2.46	2.54	2.55		2.75	2.82	2.85		1.02	1.02	0.97		0.007	0.001	0.050	
	(0.6,0.8)	10	2.55	2.54	2.57		2.60	2.69	2.65		0.85	0.90	0.83		0.021	0.051	0.024	
	(0.1,0.2)	50	2.52	2.54	2.57		2.74	2.68	2.69		0.82	0.81	0.75		0.010	0.023	0.022	
	(0.3,0.5)	50	2.56	2.57	2.58		2.75	2.65	2.74		0.51	0.52	0.47		0.004	0.013	0.007	
	(0.1,0.2)	100	2.55	2.56	2.58		2.60	2.65	2.76		0.59	0.59	0.53		0.018	0.013	0.009	
4	(0.1,0.2)	10	2.63	2.88	2.88	2.16	3.33	3.66	3.45	3.37	1.12	1.14	1.10	1.20	0.039	0.030	0.048	0.039
	(0.3,0.5)	10	2.88	2.76	2.80	2.92	2.95	3.21	3.37	3.13	0.85	0.99	1.03	0.95	0.023	0.058	0.073	0.082
	(0.6,0.8)	10	2.96	2.96	2.95	2.96	3.00	2.90	3.02	2.94	0.73	0.75	0.78	0.76	0.045	0.006	0.062	0.074
	(0.1,0.2)	50	2.91	2.87	2.87	2.93	2.91	3.00	3.31	3.15	0.63	0.79	0.84	0.75	0.006	0.109	0.099	0.093
	(0.3,0.5)	50	2.98	2.97	2.97	2.98	2.91	3.02	3.27	2.90	0.39	0.48	0.51	0.45	0.006	0.062	0.101	0.006
	(0.1,0.2)	100	2.96	2.96	2.97	2.97	2.83	2.91	3.02	2.91	0.55	0.52	0.53	0.47	0.000	0.006	0.062	0.006

## Application

In the field of ecology, the Shannon index is known alternatively as - Shannon’s diversity index, the Shannon-Weiner index, and the Shannon entropy [12]. This index was coined by Claude Shannon to the problem of textual presentation of the strings for quantification of the entropy [13]. Conceptually, it was thought when there is occurrence of different alphabets and approximately equitable distribution of the relative abundances in the textual presentation, there is less likelihood of correctly predicting appearance of next string. Consequently, there is a phenomenon of randomness. Likewise, in the field of biology too we do have variation in the phenomenon of interest. Biological communities vary in the number of species that are present and this number of species corresponding to that community is defined as richness of species. In addition to richness of a species in a community, information about the relative abundant species is also important. It is a well-known that the value of a diversity index increases when the number of types of species increases and their evenness increases. For example, communities with many species that are evenly distributed are the most diverse and communities with few species that are dominated by one species are the least diverse. By considering this distribution we expect to achieve the exact level of quantification. This index has not been used in the field of drug use epidemiology to date. Hence, we thought of applying this index concerning substance use behaviour among the street children by considering the age and the geographical variation. Our logic was that since diversity index is reflective of the number of different species and how evenly they are distributed, what incremental knowledge is achievable with respect to the substance using street children, where the correct prediction of finding any age-specific adolescent across different geographical areas is an onerous task. In this study, an attempt has been made to explore the diversity index of district viz., Central Delhi, East Delhi, New Delhi, North East Delhi, North West Delhi, South Delhi, South East Delhi, South West Delhi and West Delhi, substance use among street children in Delhi. The diversity across age-wise in these areas under the classical and Bayesian paradigms has also been evaluated. The sample size, inclusion criteria,implementation of RDS, seed plan, process of recruitment, management of coupons and incentives, data collection and ethical considerations can be found from our previous publication [14]. In this investigation due to the non-availability of the estimated size of street children across all the nine districts of Delhi, the district level representative figure of substance using street children could not be provided. The variation across different districts of Delhi is essential for developing local level intervention strategies. Hence, by applying theoretical concept of SDI, we attempted to provide more precisely the variation in the age profile of the street children.

## Results

Shannon measure is based on logarithmic scale and usually expressed in 2, 10 and e bases. For the computational purpose, we considered logarithm with base 2 in Eq. (1) for estimation of SDI of substance use under both classical and Bayesian paradigms. Here, the diversity index less than 1 denotes the low diversity and thereby low likelihood of finding substance using substance street children. Here, we have compared both classical and proposed Bayesian estimates of SDI of street children among three age groups *viz.,* 7-10, 11-14 and 15-18 years, in nine areas of Delhi in Table 2. Table 2 depicts both age-wise and district wise street children. The range of SDI estimates, viz. $\hat {H}(\boldsymbol {p}),\hat {H}(\boldsymbol {p})^{B}$, under classical(0.86-1.57) and Bayesian(0.9-1.57) set-ups were close. This implies that across all regions of Delhi the likelihood of locating substance using street children cannot be undermined. On the other hand, in Central, Eastern, Southern, Western, North East and North Western regions of Delhi, both the estimated diversity indices values were found closer to 1.5, and South East and South West regions of Delhi were closer to 1.3. In case of age-wise diversity among children, the classical estimates for all three age groups *viz.,* 7-10, 11-14 and 15-18 years, was detected to be closure to 2.9. Whereas the Bayesian estimates exhibited more diversity in the index among age-group 7-10 years (3.74) then 11-14 years (3.42) children and comparatively less among 15-18 years children (3.20). A systematic pattern was also observed among the Bayesian derived SDI among age-groups, which was not observed in case of classical SDI estimates.

**Table 2:** Classical and Bayesian estimates of Shannon diversity index (SDI) area-wise and age-class wise substance use among children in Delhi

Area	Age classes (in years)			Classical		Bayesian	
	7-10	11-14	15-18	$\hat {H}(\boldsymbol {p})$	$V\left (\hat {H}(\boldsymbol {p})\right)$	$\hat {H}(\boldsymbol {p})^{B}$	$V\left (\hat {H}(\boldsymbol {p})^{B}\right)$
Central Delhi	2	2	3	1.51	1.2023	1.51	0.0106
East Delhi	10	12	8	1.56	0.9388	1.57	0.0005
New Delhi	0	14	35	0.86	0.5153	0.90	0.0111
North East Delhi	16	35	16	1.47	0.6123	1.49	0.0046
North West Delhi	7	19	13	1.47	0.8007	1.56	0.0005
South Delhi	11	14	8	1.54	0.8953	1.56	0.0007
South East Delhi	2	13	8	1.29	0.8988	1.28	0.0042
South West Delhi	7	36	35	1.34	0.5482	1.29	0.0047
West Delhi	14	20	16	1.56	0.7579	1.57	0.0001
$\hat {H}(\boldsymbol {p})$	2.74	2.92	2.84				
$V\left (\hat {H}(\boldsymbol {p})\right)$	0.6847	0.4337	0.4659				
$\hat {H}(\boldsymbol {p})^{B}$	3.73	3.42	3.20				
$V\left (\hat {H}(\boldsymbol {p})^{B}\right)$	0.0644	0.0174	0.0271				

## Discussion

In the field of drug use epidemiology, the methodological challenges are inevitable [15]. Numerous studies have been performed in several countries like Bangladesh [16]; Pakistan [17]; Nepal [18]; Tehran [19]; Kenya [20]; Brazil [21] and India [22], but majority of them utilized purposive sampling for recruitment of street children. The lack of representativeness, has limited utility as these programs, for prevention and treatment intervention for substance using street children. There is a dearth of literature on this vulnerable population (street children) with very few studies from the developing countries. One of our previous study [14] was the first study from India that estimated the size of substance using street children in a representative manner by utilizing the Respondent Driven Sampling (RDS) survey. Hence, we attempted to move further for better characterization of the diversity. In that study we did mentioned that district level size estimation of the substance using street children could not be obtained because of the lack of count of district wise street children. Hence, under our present work we attempted the posterior characterization of the SDI by applying the figures obtained in our previous study for getting fresh insights using simulation strategies.

It is emphasized that the diversity indices estimated for street children, corresponding to each regions of Delhi, under both classical and Bayesian paradigms were close to one another, but the Bayesian estimates are much more precise than that of classical estimates. In case of age-wise diversity of street children, Bayesian estimates variation in richness towards substance using street children and were higher than that of classical estimates. Hence, the results indicate that Bayesian paradigm was more precise (lesser variance) than classical estimates of diversity. The phenomena of substance use among the adolescent’s age group are not uncommon. Based on our previous large epidemiological study on the assessment of pattern, profile, and correlates of substance use among children (both living at home or on the streets) in India, some deleterious figures of health consequences were observed on a sample of 4,024 children [23, 24] between the age of 5-18 years [Mean(SD): 15.6(2.1)]. It was found that approximately one-sixth of the children living at home and more than one-fourth of the street children were involved in the sexual behavior under the effects of the substance use. Additionally, one-sixth (16.9%) of the children at home and one fifth (20%) of the street children indulged in sexual behavior in exchange for either substances or money. The physical and psychological problems related to substance use were reported by one-half of the children and that substantial proportion also reported legal problems on account of their substance use. The complications [experienced tolerance (55%-63%) or withdrawals (56%-67%)] was found to be higher among street children and out-of-school children. Hence, the detrimental consequence of substance use in the adolescent age group cannot be ignored as it may have more serious ramifications on other dimensions of life such as social, physical, psychological, legal, etc. The estimates of the SDI under the classical approach was closure to 2.9 for all the three age groups however, Bayesian estimates exhibited more diversity in the index in the lower age-group [7-10 years (3.74); 11-14 years (3.42) children] and comparatively less among 15-18 years children (3.20). This finding demonstrates that the policy-makers should take cognizance of substance use even in the lower age-group while devising a mechanism for age appropriate treatment strategies program so that various facets of physical, psychological, social, legal, and, etc. can be addressed effectively, in the middle and late childhood.

The term species evenness refers to a situation where the number of species in various possible classes are approximately closer to each other, in the environment of their habitat. Mathematically it is defined as a diversity index, a measure of biodiversity which quantifies how equal the community is numerically. Hence, findings under the present study provide a deeper insight to the fact that there is greater likelihood of finding street children in Delhi, who use substances and belong to lower as well as the middle age group. The major strength of the present study is the theoretical extension of SDI, to the real world application. The novel mathematical framework, clearly demonstrates added benefit as it characterizes more specifically, the age profile of substance using street children in Delhi. The information gained under the Bayesian paradigm, demonstrates that in light of wider age range for substance using street children it is important to consider this factor for planning local level intervention targeted at ameliorating the conditions of street children in Delhi.

The limitation of the present study is that it took only two selected variable, viz., age and geographical location into consideration. Since we aimed at establishing the extension of theoretical aspects to real world data, we thought of using single variable. Moreover, age as a variable has important public health significance. From the perspective of the descriptive epidemiology the factor age holds its own importance. According to the ‘Gateway hypothesis’ [25], their is relationship between an early drug use and its progression in the later age. Hence, we considered the variable age. Age is also an important variable for devising age appropriate interventions. According to the World Health Organization (WHO), the age group 10-19 [26] constitutes adolescents. Our premise of using this age group was based on the consensus by the stakeholders involved in the implementation of the research study. The stakeholders were the personnel working in the Non-Governmental Organization (NGOs) with the street children and they opined that during the implementation phase the seed plan should allow intake of street children in these age groups. Further details in this regard can be found from our previous publication [14]. By this age grouping we were able to have three levels with equal age spacing. We considered the broad age group given by the WHO with flexibility for the lower age range to be a part of the seed plan in implementing the RDS survey and ensuring equal age spacing. The geographical area is yet another important covariate affecting the phenomena of drug use. There are different settings for the street children such as railway tracks, garages, places of religious importance, restaurants, flyovers, traffic signals, and, etc. Any local level programs need to understand the geographical spread of the phenomena so that one can devise focused prevention strategies for decreasing the harm associated due to substance use. The districts of a particular province/state are one such natural local areas for understanding and undertaking focused attention. Moreover, it was for the first time that the posterior characterization of the SDI was attempted in the drug use epidemiology and thus we thought that these two variables would be good way to initiate the model characterization under Bayesian paradigm. The future studies should include more variables and further extend this concept for more better and comprehensive characterization of the profile of substance using street children or even other populations.

## Conclusion

In the present article, we have investigated both classical and Bayesian in connection with the estimation of overall SDI in a population. The proposed Bayesian estimate of SDI is compared with their classical counterpart. In Bayesian statistics, the characterization of a parameter depends on the selection of the appropriate prior distribution for the analysis of data and the derived posterior is then used to draw the final conclusion. Here, we derived a particular prior based on the definition of SDI and the simulated result shows that Bayesian derived estimates of SDI are uniformly better than the classical estimate of SDI.

In the vulnerable population like that of the substance using street children, age is an important factor. Any intervention should be tailored around the age composition of street children. The study clearly demonstrates the utility of Bayesian paradigm of index based measure as the methodology for deeper understanding of age profile. The results demonstrates that such methodological innovation, unravel better characterization of age profile as it provides the evidence of propensity for finding substance using street children, who belongs to lower age group (7-10 years, highest measured entropy of 3.73) followed by middle age-group (11-14 years) and upper age group(15-18). In addition to that a systematic pattern was also captured through the Bayesian derived SDI among age-groups with lesser variability than classical SDI estimates, that can be utilized by policy makers. There is ample scope for further refinement in these estimate, by incorporating more covariates that have a possible role in initiating substance use among street children in developing countries like India.

## Appendix

**Proof of Theorem 2:** Let us assume that *X*_1_,*X*_2_,⋯,*X*_*S*_ are independent binomial random variables with *X*_*i*_ follows *Binomial*(*n*,*p*_*i*_), for all *i*=1,2,⋯,*S*. The Fisher’s information of the probability of abundance of the *i*^*th*^ species in the community, *p*_*i*_, using standard notation has been obtained as

12 $$\begin{array}{@{}rcl@{}} \mathcal{I}(p_{i})=\sum\limits_{y_{i}=0}^{n} \left(\frac{\delta}{\delta p_{i}} log f(y_{i}|p_{i})\right)^{2} f(y_{i}|p_{i}) \end{array} $$

and the inverse of the Fisher’s information matrix of species classified probabilities vector, say ***p***=(*p*_1_,⋯,*p*_*S*_), has given by

$$\begin{array}{*{20}l} \mathcal{I}^{-1}(\boldsymbol{p})&= \mathcal{I}^{-1}(p_{1},p_{2}\cdots,p_{S})\\&= \left[\begin{array}{cccc} \frac{p_{1}(1-p_{1})}{n} & 0 & \dots & 0 \\ 0 & \frac{p_{2}(1-p_{2})}{n} & \dots & 0 \\ \vdots & \vdots & \ddots & \vdots \\ 0 & 0 & \dots & \frac{p_{S}(1-p_{S})}{n} \end{array}\right] \end{array} $$

For the given linear function, *H*(***p***), of Shannon index in Eq. (1), the gradient of ***p*** has obtained as

$$\begin{aligned} D_{H}^{T}(\boldsymbol{p})&= \left[\begin{array}{cccccc} \frac{\partial H(\boldsymbol{p})}{\partial p_{1}} & \frac{\partial H(\boldsymbol{p})}{\partial p_{2}} & \dots & \frac{\partial H(\boldsymbol{p})}{\partial p_{a}} & \dots & \frac{\partial H(\boldsymbol{p})}{\partial p_{c}} \end{array}\right] \\&=\left[\begin{array}{cccccc} \gamma_{1} & \gamma_{2} & \dots & \gamma_{a} & \dots & \gamma_{s}, \end{array}\right] \end{aligned} $$

where *γ*_*i*_=−[*log*(*p*_*i*_)+1].

Let $\lambda _{i}=\left (\mathcal {I}^{-1}(\boldsymbol {{p}})\right)_{ii}$=(*i*^*th*^ diagonal element of $\mathcal {I}^{-1}(\boldsymbol {{p}}))$) $=\frac {p_{i}(1-p_{i})}{n}$, then

13 $$\begin{array}{@{}rcl@{}} D_{H}^{T}(\boldsymbol{p})\mathcal{I}^{-1}(\boldsymbol{p})&=&\left[\begin{array}{cccc} \gamma_{1} & \gamma_{2} & \dots & \gamma_{s} \end{array}\right] \left[\begin{array}{cccc} \lambda_{1} & 0 & \dots & 0 \\ 0 & \lambda_{2} & \dots & 0 \\ \vdots & \vdots & \ddots & \vdots \\ 0 & 0 & \dots & \lambda_{s} \end{array}\right]\\ &=&\left[\begin{array}{cccc} \gamma_{1}\lambda_{1} & \gamma_{2}\lambda_{2} & \dots & \gamma_{s}\lambda_{s} \end{array}\right] \end{array} $$

14 $$\begin{array}{@{}rcl@{}} D_{H}^{T}(\boldsymbol{p})\mathcal{I}^{-1}(\boldsymbol{p})D_{H}(\boldsymbol{p})&=&\sum\limits_{i=1}^{s}\gamma_{s}^{2}\lambda_{s}. \end{array} $$

Let us consider,

15 $$\begin{array}{@{}rcl@{}} \boldsymbol{\phi}^{T}(\boldsymbol{p})&=&\frac{D_{H}^{T}(\boldsymbol{p})\mathcal{I}^{-1}(\boldsymbol{p})}{\sqrt{D_{H}^{T}(\boldsymbol{p})\mathcal{I}^{-1}(\boldsymbol{p})D_{H}(\boldsymbol{p})}} \end{array} $$

16 $$\begin{array}{@{}rcl@{}} &=&\left[\begin{array}{cccc} \frac{\gamma_{1}\lambda_{1}}{\sqrt{\sum\limits_{i=1}^{s}\gamma_{s}^{2}\lambda_{s}}} &\frac{\gamma_{2}\lambda_{2}}{\sqrt{\sum\limits_{i=1}^{s}\gamma_{s}^{2}\lambda_{s}}} & \dots & \frac{\gamma_{s}\lambda_{s}}{\sqrt{\sum\limits_{i=1}^{s}\gamma_{s}^{2}\lambda_{s}}} \end{array}\right]\\ &=&\left[\begin{array}{cccc} \phi_{1}(\boldsymbol{p}) & \phi_{2}(\boldsymbol{p}) & \dots & \phi_{S}(\boldsymbol{p}) \end{array}\right], \end{array} $$

where, $ \phi _{i}(\boldsymbol {p})=\frac {\gamma _{s}\lambda _{s}}{\sqrt {\sum \limits _{i=1}^{s}\gamma _{s}^{2}\lambda _{s}}}=-\left [log(p_{i})+1\right ]\frac {p_{i}(1-p_{i})}{n}\left (\sqrt {\sum \limits _{i=1}^{S}\left [log(p_{i})+1\right ]^{2} \ \frac {p_{i}(1-p_{i})}{n}}\right)^{-1}$.

In context of deriving a prior distribution of a parameter, [27] has suggested the criteria that must be satisfied to establish the posterior distribution for a parametric function and is given by

$$ \sum\limits_{i=1}^{S}\frac{\partial}{\partial p_{i}} \ \phi_{i}(\boldsymbol{p}) \ q(\boldsymbol{p})=0   $$

Let

$$\begin{aligned} q(\boldsymbol{p})&=\left(\sum\limits_{i=1}^{S}\left[log(p_{i})+1\right]^{2} \ \frac{p_{i}(1-p_{i})}{n} \right)^{\frac{1}{2}}\\&\quad \prod\limits_{i=1}^{S} \left[p_{i}(1-p_{i})(1+log(p_{i}))\right]^{-1} \end{aligned} $$

then

17 $$\begin{array}{@{}rcl@{}} \phi_{1}(\boldsymbol{p}) \ q(\boldsymbol{p})&=&\frac{-1}{n}\prod\limits_{i\neq 1}^{S} \left[p_{i}(1-p_{i})(1+log(p_{i}))\right]^{-1}\\  \Rightarrow \ \frac{\partial}{\partial p_{1}} \phi_{1}(\boldsymbol{p}) \ q(\boldsymbol{p})&=& 0 \end{array} $$

and

18 $$\begin{array}{@{}rcl@{}} \phi_{j}(\boldsymbol{p}) \ q(\boldsymbol{p})&=&\frac{-1}{n}\prod\limits_{i\neq j=1}^{S} \left[p_{i}(1-p_{i})(1+log(p_{i}))\right]^{-1}\\  \Rightarrow \ \frac{\partial}{\partial p_{j}}\phi_{j}(\boldsymbol{p}) \ q(\boldsymbol{p})&=& 0. \end{array} $$

From the above Eqs. (17) and (18) we have

$$\sum\limits_{i=1}^{S}\frac{\partial}{\partial p_{i}} \ \phi_{j}(\boldsymbol{p}) \ q(\boldsymbol{p})=0, $$

which satisfied the condition required to be a prior distribution, *q*(***p***), of a parameter. Therefore,

$$\begin{aligned} q(\boldsymbol{p})&\propto \left\lbrace\sum\limits_{i=1}^{S}\left(1+log(p_{i})\right)^{2} \ p_{i}(1-p_{i})\right\rbrace^{\frac{1}{2}}\\& \quad\prod\limits_{i=1}^{S} \left[p_{i}(1-p_{i})(1+log(p_{i}))\right]^{-1} \ ; \ 0< p_{i} < 1 \end{aligned} $$

and hence we get the required proof.

## Metropolis-Hastings algorithm

The basis for opting Metropolis-Hastings (MH) algorithm is to simulate samples from a probability distribution by using the full joint density function and (independent) proposals distributions for each variable of interest. The steps followed under this algorithm consist of three components namely, propose a proposal distribution to generate sample, computation of the acceptance probability, acceptance criteria of a sample generated through the proposal distribution that are given below:

**Initialize**Initialize the sample value, say *π*, for each random variable from *g*(.)∼*Uniform*(0,1).

$$\pi^{(0)} \ \ \uparrow \ \ g(\pi)$$

**for** iteration *i*=1,2,⋯ do {Propose:Generate a proposal (or a candidate) sample *π*^*cand*^ from the proposal distribution *q*(*π*^(*i*)^|*π*^(*i*−1)^)

$$\pi^{cand} \ \ \uparrow \ h\left(\pi^{(i)}|\pi^{(i-1)}\right)$$

Acceptance Probability:Compute the acceptance probability via the acceptance function *α* (*π*^*cand*^|*π*^(*i*−1)^) based on the proposal distribution and the full joint density *g*(.)

$$\begin{array}{@{}rcl@{}} \alpha \ \left(\pi^{cand}| \pi^{(i-1)}\right)=Min \ \left\lbrace 1,\, \frac{ h\left(\pi^{(i)}|\pi^{cand}\right) g\left(\pi^{cand}\right)}{h\left(\pi^{cand}|\pi^{(i-1)}\right) g\left(\pi^{(i-1)}\right)}\right\rbrace  \end{array} $$

Generate *u* ∼ Uniform (*u*;0,1)**if** *u*<*α***then**Accept the proposal: *π*^(*i*)^←*π*^*cand*^**else**Reject the proposal: *π*^(*i*)^←*π*^(*i*−1)^**end if**Accept the candidate sample with probability *α*, the acceptance probability, or reject it with probability 1−*α***end for** }

## Data Availability

The data and the material are under the Government Jurisdiction and if sought necessary to be made available, need the permission for the same through the competent authority. In this reference the corresponding author can be contacted.

## Abbreviations

SDI: Shannon’s diversity index
RDS: Response driven sampling
MCMC: Markov Chain Monte Carlo
